# Progress in the Mechanism of Autophagy and Traditional Chinese Medicine Herb Involved in Dementia

**DOI:** 10.3389/fphar.2021.825330

**Published:** 2022-02-15

**Authors:** Pengyu Tao, Jing Ji, Simeng Gu, Qian Wang, Yuzhen Xu

**Affiliations:** ^1^ Basic Medical School, Shanghai University of Traditional Chinese Medicine, Shanghai, China; ^2^ Department of Nephrology, Yueyang Hospital Affiliated to Shanghai University of Traditional Chinese Medicine, Shanghai, China; ^3^ Department of Psychology, Jiangsu University Medical School, Zhenjiang, China; ^4^ Postdoctoral Workstation, Department of Central Laboratory, Taian City Central Hospital, Shandong First Medical University and Shandong Academy of Medical Sciences, Taian, China; ^5^ Department of Rehabilitation, The Second Affiliated Hospital of Shandong First Medical University, Taian, China

**Keywords:** autophagy, Chinese herbal medicine extracts, dementia, inflammation, oxidative stress, apoptosis

## Abstract

Dementias is a kind of neurodegenerative disease, which occurs among the aging population. Current therapeutic outcome for dementia is limited. The medical use of herbal plant has a rich history in traditional Chinese medicine practice for thousands of years. Herbal medicine (HM) may provide a positive effect for prevention and treatment in dementia. As an alternative treatment to dementia, there has been a growing interest in HM extracts in scientific community as a result of its promising study results, mainly in animal experiment. At the molecular level, HM extracts trigger autophagy and reduce generation of reactive oxygen species (ROS) while inhibiting inflammation and reduce neurotoxicity. Experiments both *in vivo* and *in vitro* have identified certain potential of HM extracts and natural products as an important regulator factor in mediating autophagy, which might contribute to the improvement of dementia. This brief review not only summarizes the mechanism of autophagy in dementia but also offers a general understanding of the therapeutic mechanism of HM extracts in treating dementia and evaluates the potential clinical practice of HM in general.

## Introduction

Dementias, mainly including Alzheimer’s disease (AD) and vascular dementia (VaD) (the two major form of dementia), are characterized by memory loss, damaged judgment, and language barrier seriously affecting daily life (Xu et al., 2019). It is estimated by the WHO report based on epidemiological data that the number of people across the world suffering from dementias will jump to 81.1 million by 2040 (Le Couteur et al., 2020). It is universally acknowledged that heart attack, stroke, atherosclerosis, hypertension, obesity, smoking, and cardiac problems are vital risk factors contributing to the cause of vascular dementia (Sahyouni et al., 2021). Clinically, dementia covers a wide range of brain abnormalities, ranging from slow progressive loss of memory, cognitive function, to a failure to conduct personal daily activities. The hallmark of dementia is characterized by accumulation of β-amyloid (Aβ) and hyper-phosphorylated Tau protein. The overexpression of these hallmarks often indicate neurodegenerative disorders that may turn into severe pathology. Thus, targeting these hallmarks may relieve the symptoms of dementias on the whole, and triggering autophagy by HM extracts is likely to be an alternative treatment for dementias (Padilla, 2019; Paik et al., 2020).

Autophagy has been interfered in the pathogenesis of dementias (He and Klionsky, 2009). It is generally assumed that autophagy is activated and acts as a protective mechanism in response to stimulating factors, thus attenuating toxic damage to the neuron. A variety of methods can be the promoter of autophagy, such as less food intake, regular sport, rapamycin, and AMP-activated protein kinase (AMPK)-activated protein kinase (Papandreou et al., 2008; Wang et al., 2021). However, once the autophagy pathway is impaired by stroke, atherosclerosis, and hypertension, this might lead to the progressive accumulation of toxic proteins in neuron, which finally contribute to the development of VaD (Romeo et al., 2019). Moreover, the activation of autophagy plays a key role in protecting and maintaining vascular integrity, and inhibition of autophagy is acting as a negative regulator in vascular degeneration, aging, and related pathological conditions (Levine and Kroemer, 2008; Ju and Weihl, 2010; Wang et al., 2021).

The incidence rate and mortality of dementias experienced a fast growth year by year. Current therapies simply offer symptomatic relief and fail to achieve a desired efficacy against dementias. Thus, there is an urgent need to seek an alternative treatment (Levine and Kroemer, 2008). Traditional Chinese medicine (TCM) is rooted in yin-yang theory and has been broadly used for the prevention and treatment of neurodegenerative disease in China for centuries (Han et al., 2012; Wang et al., 2021). As some Chinese medicines act as alternative approach in treating dementia and show effects on relieving dementia-related syndrome (Bai and Zhang, 2021), it has drawn great attention among researchers. It is the plant elements or extracts that carry out therapeutic function (Li and Zhang, 2009). The derivations of medicinal herbs have shown potential in alleviating dementia; the herb-plant-derived natural products tested in clinical trials have obtained positive results of improving dementias (Lin et al., 2017). Recently, studies have suggested that medicinal herb extracts could slow down the development of dementia *via* activating different pathways, which might provide a guideline of TCM for VaD therapy (Shen and Li, 2005; Zhu et al., 2006).

## The Role of Autophagy and Its Related Signaling Pathway in Dementia

**FIGURE 1:**
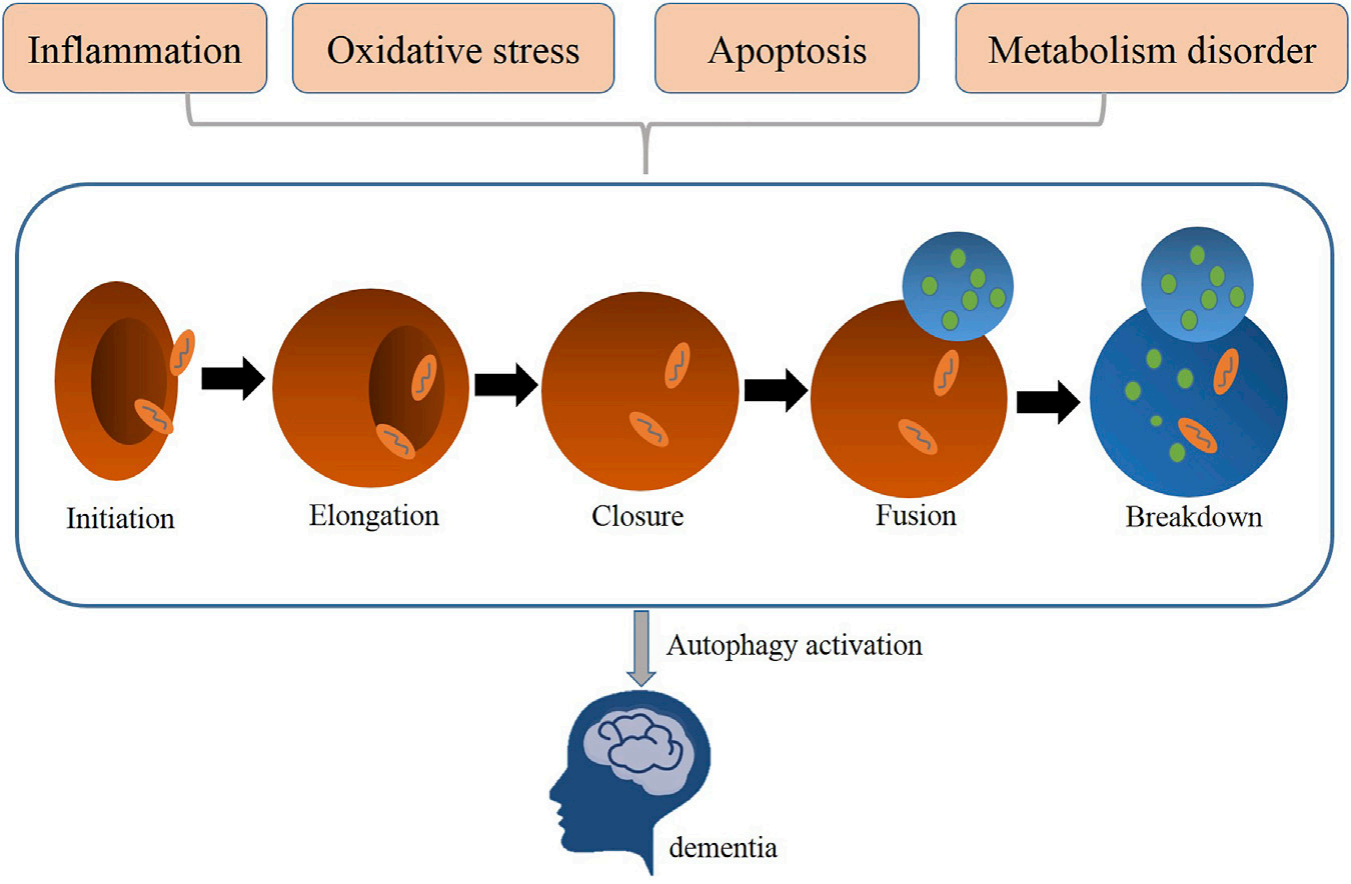
Related pathological processes (metabolism disorder, oxidative stress, apoptosis, inflammation) mediated autophagy in dementia.

Autophagy is a highly conserved self-digestion process involved in physiology to maintain cellular homeostasis *via* delivering dispensable or potentially damaged intracellular components, such as proteins and organelles to lysosomes for degradation, clearance, and recycling (He and Klionsky, 2009). Macroautophagy, microautophagy, and chaperone-mediated autophagy are three main forms of autophagy, all of which vary from their mechanisms to functions (Morita et al., 2010). Of these three types, macroautophagy is the most intensively studied autophagy process (Wang et al., 2020a); thus, it is generally referred to as autophagy. Autophagy is a normal physiological activity occurring in most cells to maintain homeostasis. But under some extreme situation, autophagy is usually activated to act as an adaptive and protective mechanism in alleviating damage of cells exposed to risky factors, such as oxidative stress, inflammation, or metabolic disorders (Morita et al., 2010). Researchers found that the dysfunction of autophagy contributes to the pathogenesis of neurodegenerative disease, such as vascular dementia (Figure 1). Thus, restoring autophagy activation may improve the prognosis of dementia (Cuervo, 2013; Caberlotto and Nguyen, 2014).

The mammalian neuron system depends on autophagy to maintain its normal functions and homeostasis (Jiang and Chang, 2019). Numerous specific-gene knockout animal models have been established to show that deletion of core autophagy-related genes leads to neonatal and embryonic lethality (Alirezaei et al., 2008). Three major steps are necessary for the formation of autophagosome: initiation, nucleation, and elongation. With more than 30 autophagy-related genes (ATGs) participating in autophagy activation, this complex process is strictly regulated to keep dynamic balance between synthesis and degradation, use, and recycling of intracellular substances (Caberlotto and Nguyen, 2014). The ULK1/2–ATG13–FIP200–ATG101 complex is important for activating autophagy. The Becl-1–PI3K complex is necessary for the phagophore nucleation step. The Atg12–Atg5–Atg16 complex and LC3/Atg8 cascade are employed in the autophagosomes elongation step (Cuervo, 2013; Liu et al., 2014; Nussenzweig et al., 2015). The conversion of LC3-I to LC3-II is recognized as a marker of autophagy activation and autophagosome formation (Levine and Kroemer, 2008).

mTOR is the best known mammalian target of rapamycin and also considered one of the most significant autophagy regulators in eukaryotic cells that includes two mTOR complexes, the mTOR complex 1 (mTORC1) and complex 2 (mTORC2); mTORC1 is the most extensive studied complex (Cao, 2010). Under most normal conditions, mTORC1 suppresses autophagy function *via* phosphorylating ULK1 and thereby inhibiting its activity (Alers et al., 2012). However, once cells are exposed to oxidative stress stimuli, the autophagy process can be initiated *via* the inactivation of mTORC1 (Kim et al., 2011). In the nervous system, the autophagy function can be neuroprotective *via* the inhibition of mTORC1. The high expression of mTORC1 is frequently observed in animals with nervous system diseases, such as vascular dementia (Guo et al., 2021; Perluigi et al., 2021). Thus, autophagy activation with the inhibition of mTOR expression can lead to neural tissue protection (Cao, 2010). Results from experimental models of AD indicated that disease progression seems to have a connection with dysfunctional autophagic processes and be improved by inhibition of mTOR activity (Joungmok and Kun-Liang, 2019).

Sirtuin1 (SIRT1), a homolog of Sir2, regulates energy metabolism, longevity, and autophagy in an NAD+-dependent manner (Ji et al., 2020). The sirt1 activity promotes life survival in organisms and protects neuronal cells from oxidative stress (Salminen and Kaarniranta, 2009). SIRT1 is responsible for the homeostasis of neural systems (Kim et al., 2007). If sirt1 activity is lost, this leads to impaired cognitive abilities, elevated Aβ production, and accumulation in AD patients (Qin et al., 2006). The increased sirt1 level can induce autophagy *via* prohibiting mTOR pathways to protect cells exposed to oxidative stress (Preeti et al., 2017). Overexpression of sirt1 can promote nerve cell growth and regulate cellular metabolism by suppressing mTOR level (Süleyman et al., 2012). The enhanced SIRT1 activity could reduce the toxic damage imposing on nerve cells by activating AMPK-mediated autophagy process, which is essential for cell survival (Cantó et al., 2009; Dong et al., 2015). The sirt1-mediated autophagy may represent a promising therapeutic treatment to block the neurodegenerative disease (Kilic et al., 2018).

## TCM for Dementia

**FIGURE 2:**
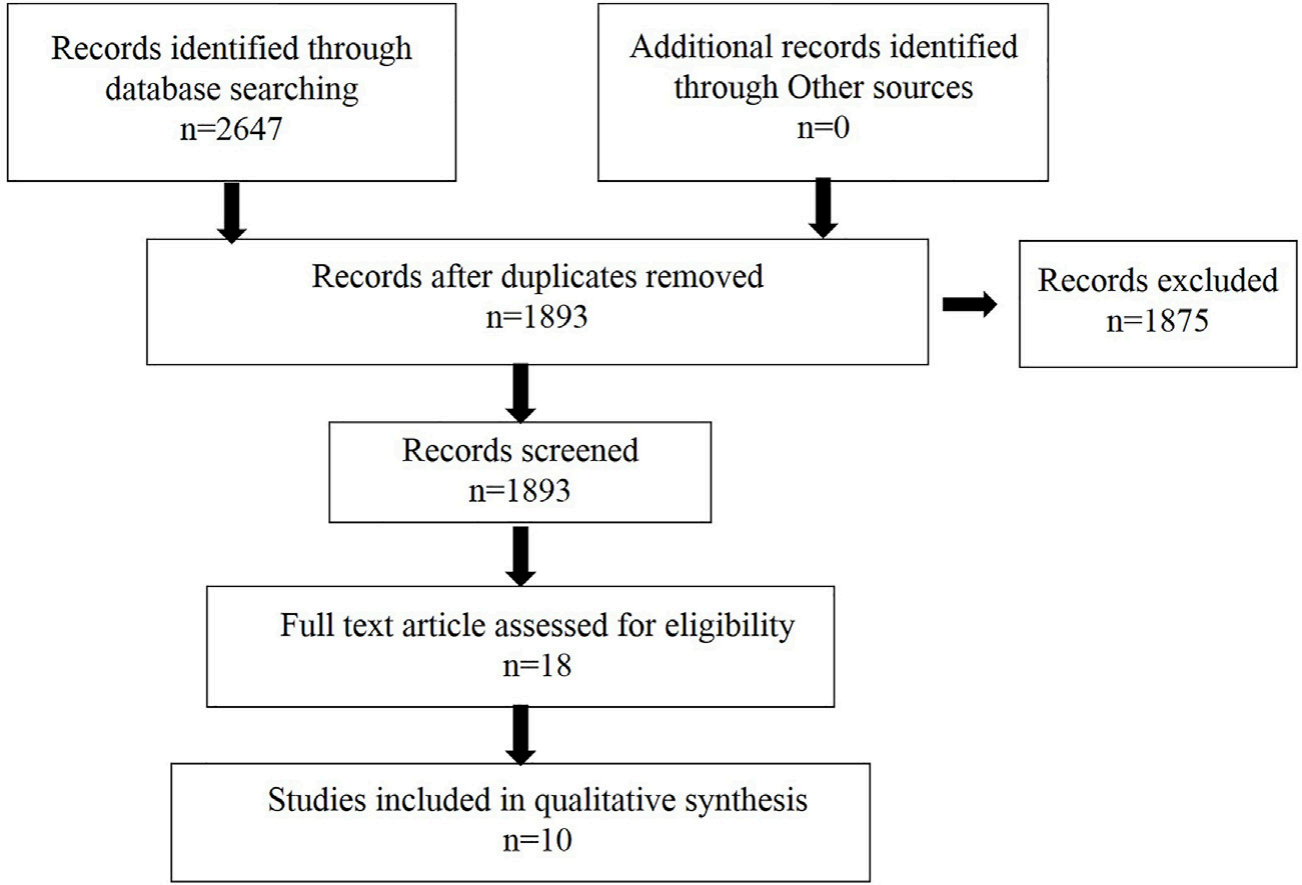
Flow chart of literature research used to retrieve information to identify potential extracts for dementia treatment.

**FIGURE 3:**
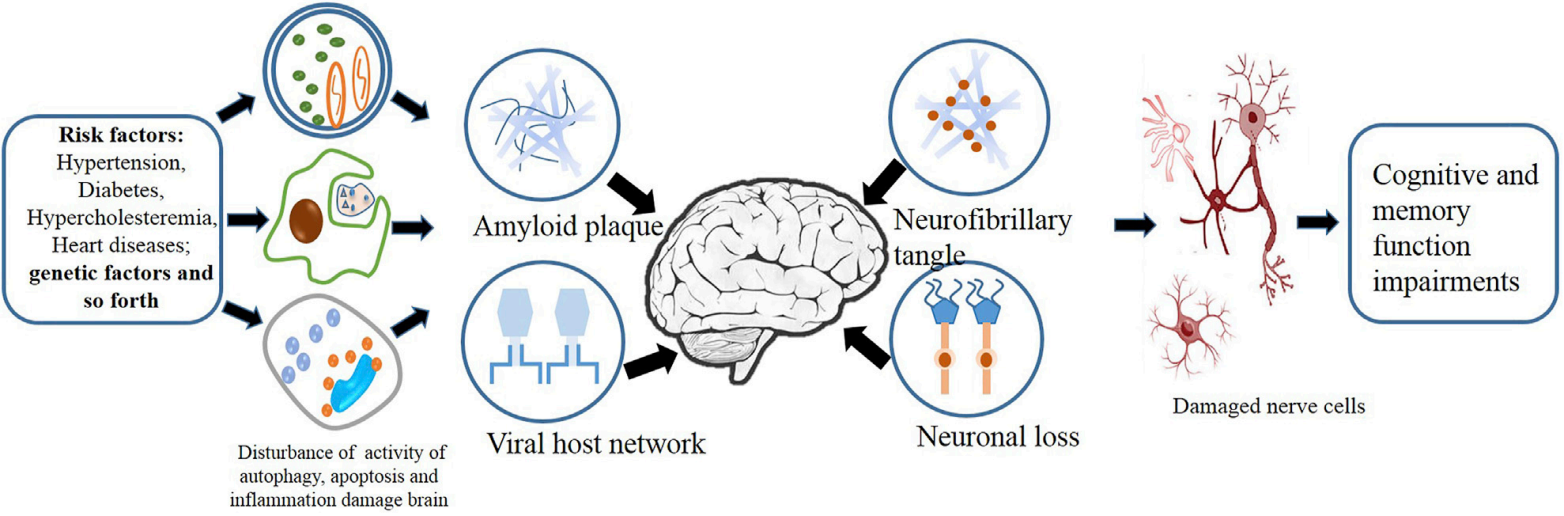
Factors including inflammation, autophagy, and apoptosis act on the complex cellular network of the brain.

We searched the relevant literature to determine the extracts that may be used to treat dementia. The flow chart is shown in Figure 2, and the specific extracts are summarized in Table 1. The cholinesterase inhibitors approved by FDA have been put into clinical use for relieving the symptoms of dementia rather than therapeutic drugs (Eglit, 1999). Patients with progressive dementia may need to take more doses of cholinesterase inhibitors than before, which bring some unwanted effects, such as nausea, vomiting, and diarrhea; these adverse events limit its clinical treatment (Skaljo et al., 2009; Lu and Herr, 2012). The combination multi-targets therapy is essential to slow down the progression of the pathogenesis of dementia from multiple perspectives (Li, 2018). TCM has become a hot point in research due to its representative of multiple targets and multiple pathway, and they play a significant role in discovering new drugs against dementia. The combination and application of various herbs are the core heart of TCM, and this process is complex, which produces different outcomes, since the complexity of formulas often reacts to different targets or pathways (Figure 3). In order to get a satisfactory efficacy, some bioactive extracts derived from herbal plants have already shown promising results in study on dementias (Ma et al., 2019; Wang et al., 2020b). In this study, we will summarize some protective function of active extracts isolated form Chinese herbal plan on dementia.

**TABLE 1:** Herbal medicine exerts protective effect on dementia.

Herb name	Effective ingredients and protective mechanism
Salviae Miltiorrhizae	Tanshinone IIA blocking the expression of iNOS and MMP-2 protein and reduce the production of ROS (Kong et al., 2021)
Mulberry	Resveratrol inhibit inflammation *via* decreasing expression of IL-1β, IL-6, and TNF-α (Zhao et al., 2019)
Cortex *Phellodendron amurensis*	Ethanol extract of Cortex *Phellodendron amurensis* promote the survival of nerve cells under toxic situation by increasing the level of Bcl-2/Bax protein (Xian et al., 2013)
*Ganoderma lucidum*	*Ganoderma lucidum* triterpenoids attenuated neuronal damage through decreasing the expression of antioxidative protein Nrf2, HO1, and NQO1 (Yu et al., 2020)
*Curcuma longa*	Curcumin improved spatial learning and memory *via* upregulating the expression of IGF-1R, IRS-2, and Akt (Natascia et al., 2014)

## HM Extracts on Oxidative Stress

Numerous studies have shown that the overgeneration of reactive oxygen species and inactive antioxidant defenses result in the failure in removing free radicals or fixing organ damage that leads to the occurrence of oxidative stress, which is considered as a key role in the pathogenesis of dementias (Mao, 2012).

Salviae Miltiorrhizae is a popular Chinese herbal plant that is widely employed in the treatment of nerve system disease including dementia (Paudel et al., 2020). Tanshinone IIA (Tan IIA) is an active compound isolated from Salviae Miltiorrhizae (Kong et al., 2021). It is reported that Tan IIA could effectively delay the progression of AD in rats through blocking the expression of inducible nitric oxide synthase (iNOS) and matrix metallopeptidase 2 (MMP-2) protein and also improve memory function and learning ability of rats with AD *via* reducing the generation of ROS contributed to oxidative stress (Zhi et al., 2009; Jiang et al., 2014; Tang et al., 2017).

Rehmanniae Radix is one of the commonly used Chinese herbal medicine, which could nourish kidney yin to make the brain clearer based on Chinese medicine theory (Sun et al., 2014). Rehmannioside A (ReA) is a main compound isolated from Rehmanniae Radix, which offers protection against various diseases (Jung et al., 2013). AD rats treated with Rehmannioside A have shown a great improvement in cognitive function and memory by promoting neuro growth; the mechanism is partially associated with the inhibition of NOS and superoxide dismutase (SOD) activity (Sun et al., 2014; Sun et al., 2019).

## HM Extracts on Apoptosis

The integrity of nerve cell is vital to the normal functional activities of the nerve system (Heydarabadi et al., 2020). Thus, progressive accumulation of impaired nerve cell will inevitably lead to a series of neurological dysfunction (Wang et al., 2019). Recent studies indicate that oxidative stress and metabolic disorder are related to the apoptosis of nerve cells, which is the main factor responsible for the neuron loss in patients with dementia. Handling apoptosis is recognized as an effective therapy in alleviating dementia (Zhang et al., 2019).

Fucoidan is a natural polysaccharide mainly isolated from brown algae with various bioactive functions, including anti-inflammatory (Lee et al., 2012). A study indicated that fucoidan could protect nerve cell survival from being exposed to oxidative stress through inhibiting the expression of capase-3, which is considered to be vital role in the regulation of apoptosis (Subaraja et al., 2020).

Cortex *Phellodendron amurensis* (CPA), also known as “Huang Bai,” is employed in Chinese medicine clinical practice as anti-heat herb for relieving inflammatory conditions and calming spirit (Xian et al., 2011). The ethanol extract of Cortex *Phellodendron amurensis* shows great potential in reducing neurotoxicity in beta-amyloid (Aβ)-induced PC12 cells. The mechanism by which the ethanol extract of CPA affects the survival of PC12 cells are likely to be associated with increasing the level of Bcl-2/Bax protein and decreasing the level of capase-3 protein so as to inhibit the occurrence of nerve cell apoptosis and exert neuroprotective effect against toxicity (Xian et al., 2013).

Salviae Miltiorrhizae-derived Tanshinone IIA (Tan IIA) exhibited a strong potential to ameliorate beta-amyloid peptides (Aβ)-induced cytotoxicity in PC-12 cells, which is generally used as AD research model (Dong et al., 2012). Treatment of Tan IIA in dementia is capable of protecting the brain under chronic injury and inhibiting apoptosis occurring among cells, with a repression of PI3K/Akt pathways and a down-level of anti-autophagic regulator mTOR (Dong et al., 2012).

## HM Extracts on Inflammation

Inflammation observed in the development of dementia is recognized as a typical pathological feature (Lee et al., 2020). The beta-amyloid peptides (Aβ)-induced inflammation in nerve cells causes the neuron loss and cognitive impairment. The injured nerve cells with low autophagy activity produce proinflammatory cytokines and chemokines, which further enhance the activity of inflammation (Lee et al., 2020). Targeted inflammation and restoration of autophagy activity could be a promising therapy in alleviating dementia.

Dioscin is an active compound derived from Polygonatum Zanlanscianense Pamp. Dioscin could remarkably prohibit the beta-amyloid peptides (Aβ)-induced neurotoxicity in animals with dementia and the decline in number of apoptotic cell and reactive oxygen species (ROS) production (Zhang et al., 2020). The mechanism is linked to the suppression of inflammation by downregulation of interleukin (IL)-1β, IL-6, and tumor necrosis factor alpha (TNF-α) protein. In addition, further findings indicated that the administration of Dioscin upregulates the expression of Beclin-1 and LC3-II level and sequentially restore autophagy activity (Zhang et al., 2020).

Resveratrol is a polyphenolic ascorbic acid derived from plants, such as wine, apples, and peanuts, which exert a strong protective effect against inflammation (Moussa et al., 2017). The accumulation of evidence indicated that Resveratrol is capable of blocking inflammation in the brain of animals with dementia, and the reduction in expression of IL-1β, IL-6, and TNF-α (Zhao et al., 2019). In addition, resveratrol is reported to restore autophagy activity through enhancing the expression of Beclin-1 and LC3-II level and suppressing nuclear factor kappa B (NF-κB)-mediated inflammation (Fisher, 2021), suggesting that restoration of autophagy activity could be used as a treatment against inflammation-induced dementia.

## HM Extracts on Energy Dysfunction

Mitochondria is a kind of organelle serving as the energy source of cellular function. Differences in the number, structure, and enzyme activity of mitochondria are existing between AD patients and non-AD patients (Eckert, 2019). Mitochondrial dysfunction induced by beta-amyloid peptides (Aβ) has been identified as an early marker during the development of AD, mainly characterized by the brain metabolism disorder, the dysregulation of calcium homeostasis, and the rising ROS level (Guo et al., 2019).

Curcumin is an active compound derived from *Curcuma longa* and shows therapeutic potential in regulating metabolism (Isik et al., 2009). Numerous studies investigating the effect of curcumin on glucose metabolism indicated that treatment of APP/PS1 double transgenic mice with curcumin enhanced glucose uptake and ameliorated impaired insulin signaling pathway in the brain and improved spatial learning and memory *via* upregulating the expression of IGF-1R, IRS-2, and Akt protein expression (Miyasaka et al., 2010; Natascia et al., 2014). These findings lead to a conclusion that targeting metabolism-related signaling may act as an innovative method for preventing Alzheimer’s-related dementia.

Ganoderma lucidum triterpenoids (GLTs) are the major meditative compounds extracted from *Ganoderma lucidum*, which is documented in Chinese medicine therapy as a folk remedy based on its multifunctional health-promoting abilities (Yu et al., 2020). Multiple studies on neuroprotective effect of GLTs implied that GLTs improved cognitive impairment, attenuated nerve damage, and suppressed cell apoptosis in the nerve cells in AD animals through decreasing expression of anti-oxidative protein Nrf2, HO1, and NQO1 to inhibit the generation of ROS in the brain and promote nerve cell survival under oxidative stress situation (Yu et al., 2020).

## Conclusion and Perspective

**FIGURE 4:**
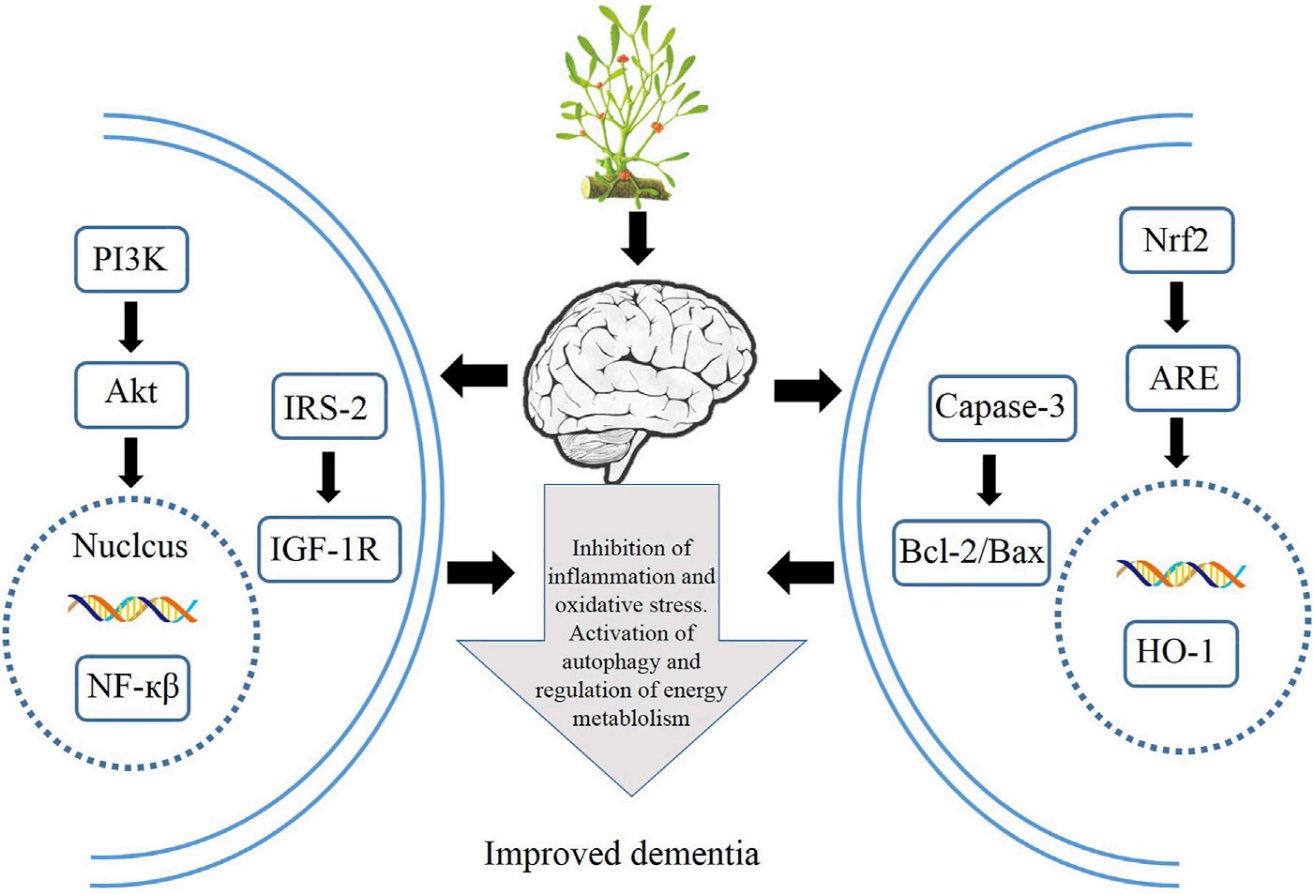
Therapeutic applications of HMs *via* modulation of autophagy, inflammation, oxidative stress, and energy metabolism.

HM extracts have shown its great efficacy in improving dementia, although the mechanisms are still under exploration. Recent findings highlight the role of HM extracts *via* activating various signaling pathways to attenuate cognitive barrier, spatial learning, and energy metabolism in both animal experiments and cell lines. The generally used HM extracts, such as resveratrol, Tanshinone IIA, dioscin, and curcumin, are potential candidates for new drug screening against dementia, and numerous results have demonstrated their therapeutic efficacy to attenuate nerve cell damage in both animal mode and cell lines *via* activating autophagy and anti-inflammation and inhibiting apoptosis. The relevant mechanisms of the therapeutic application of HMs are summarized in Figure 4. Although these results sounds promising, further studies into the safety of HM extracts should be carried out, since multiple targets are involved, and most of the results are obtained from experiments; clinical practices are in great need to assess the therapeutic efficacy and safety on the human body. Therefore, HM extracts offer an alternative solution to the treatment of dementias *via* mediating various signaling pathways.
